# Novel deep learning-based prediction of HER2 expression in breast cancer using multimodal MRI, nomogram, and decision curve analysis

**DOI:** 10.3389/fonc.2025.1593033

**Published:** 2025-10-29

**Authors:** Shi Qiu, Qianqian Zhao, Yun Zhao

**Affiliations:** ^1^ Department of Oncology, Affiliated Hospital of Jiangnan University, Wuxi, Jiangsu, China; ^2^ Echocardiography Department, The Second Affiliated Hospital of Shandong First Medical University, Tai’an, Shandong, China

**Keywords:** HER2 status, breast cancer, deep learning, MRI sequences, clinical data, imaging biomarkers, nomogram, feature selection

## Abstract

**Objective:**

This study aimed to develop a robust, automated framework for predicting HER2 expression in breast cancer by integrating multi-sequence breast MRI with deep learning-based feature extraction and clinical data. The goal was to improve prediction accuracy for HER2 status, which is crucial for guiding targeted therapies.

**Materials and Methods:**

A retrospective analysis was conducted on 6,438 breast cancer patients (2006–2024), with 2,400 cases (1,286 HER2-positive, 1,114 HER2-negative) selected based on complete imaging and molecular data. Patients underwent 3T MRI scans with T1, T2, and contrast-enhanced (DCE) sequences. Imaging data from four medical centers were standardized through preprocessing steps, including intensity normalization, registration, and motion correction. Deep learning feature extraction utilized ResNet50, VGG16, EfficientNet-B0, and ViT-Small, followed by ICC filtering (≥0.9) and LASSO regression for feature selection. Nomogram construction, ROC analysis, and DCA evaluation were performed to assess model performance. Statistical analyses were conducted using Python and R, with significance set at p < 0.05.

**Results:**

In this study, we developed an integrated predictive model for HER2 status in breast cancer by combining deep learning-based MRI features and clinical data. The model achieved an AUC of 0.94, outperforming traditional methods. Analysis revealed significant differences between HER2-positive and HER2-negative groups in tumor size, lymph node involvement, and microcalcifications. Imaging features, such as washout enhancement and peritumoral edema, were indicative of HER2 positivity. After applying ICC filtering and LASSO regression, the selected features were used to construct a nomogram, which demonstrated strong predictive accuracy and calibration. The DCA confirmed the model’s clinical utility, offering enhanced decision-making for personalized treatment.

**Conclusions:**

This study demonstrates that integrating deep learning with multi-sequence breast MRI and clinical data provides a highly effective and reliable tool for predicting HER2 expression in breast cancer. The model’s performance, validated through rigorous evaluation, offers significant potential for clinical implementation in personalized oncology, improving decision-making and treatment planning for breast cancer patients.

## 1 Introduction

Breast cancer is a heterogeneous disease with multiple molecular subtypes, among which the overexpression of human epidermal growth factor receptor 2 (HER2) plays a crucial role in prognosis and treatment strategies. HER2-positive breast cancer, characterized by the amplification of the ERBB2 gene, accounts for approximately 15–20% of all breast cancer cases and is associated with aggressive tumor behavior and poor prognosis (1, 2). Accurate determination of HER2 expression is essential for guiding targeted therapies, such as trastuzumab and pertuzumab, which have significantly improved patient outcomes. Traditional methods for HER2 evaluation, including immunohistochemistry (IHC) and fluorescence *in situ* hybridization (FISH), are labor-intensive and prone to interobserver variability, necessitating more objective and automated approaches for precise HER2 classification (3).

Deep learning has emerged as a powerful tool in medical imaging, capable of extracting high-dimensional, discriminative features from radiological data. Unlike conventional radiomics, which relies on handcrafted features, deep learning models autonomously learn hierarchical representations, improving classification performance (4, 5). In this study, four deep learning architectures—ResNet50, VGG16, EfficientNet-B0, and Vision Transformer (ViT-Small)—were employed for feature extraction from multi-sequence breast MRI scans. Each model offers unique advantages: ResNet50 effectively captures complex spatial features through residual learning, VGG16 provides a structured feature hierarchy, EfficientNet-B0 optimizes computational efficiency while maintaining high accuracy, and Vision Transformer leverages self-attention mechanisms for enhanced feature encoding. These extracted deep features provide a robust basis for distinguishing HER2 expression levels in breast cancer (6).

To enhance clinical interpretability and decision-making, a nomogram was developed by integrating deep learning-derived features with statistically significant clinical variables. Nomograms offer a user-friendly graphical representation of predictive models, facilitating individualized risk assessment. Additionally, decision curve analysis (DCA) was performed to evaluate the net clinical benefit of the developed models, ensuring their applicability across different probability thresholds. Furthermore, model performance was rigorously validated using Receiver Operating Characteristic (ROC) analysis, with the Area Under the Curve (AUC) serving as a key metric for assessing discriminative ability. This comprehensive approach bridges the gap between computational modeling and practical clinical application, promoting precision oncology (5–10).

The robustness and generalizability of predictive models depend on the diversity of the study population. This study leverages a multicenter dataset spanning 6,438 patients from 2006 to 2024, ensuring a heterogeneous representation of breast cancer cases. Multicenter studies mitigate biases associated with single-institution datasets, enhance model external validity, and improve generalizability to broader clinical settings. By incorporating diverse imaging protocols, genetic backgrounds, and clinical characteristics, the findings of this study are more applicable to real-world scenarios, strengthening the reliability of the proposed methodology.

This study presents a novel, large-scale, multicenter approach to distinguishing HER2 expression levels in breast cancer using deep learning and nomogram-based predictive modeling. The key contributions of this research are:

1. First study to integrate deep learning-based feature extraction from multi-sequence breast MRI with HER2 classification using four state-of-the-art models (ResNet50, VGG16, EfficientNet-B0, and Vision Transformer).
2. Application of a comprehensive feature selection strategy, including intraclass correlation coefficient (ICC) filtering and LASSO dimensionality reduction, ensuring optimal feature extraction.
3. Development of a nomogram incorporating deep learning features and clinical biomarkers to facilitate practical implementation in clinical settings.
4. Evaluation of model performance using DCA and ROC analysis, ensuring clinical utility and reliability of the predictive framework.
5. First large-scale multicenter study (n = 6,438) on HER2 classification using deep learning and radiomics, enhancing the generalizability and robustness of results.

By integrating deep learning with clinical decision-support tools, this study advances the field of radiogenomics and personalized breast cancer management, offering a novel, automated framework for HER2 classification.

## 2 Materials and methods

### 2.1 Study design and patient population

The dataset was collected from four anonymized tertiary referral centers between 2006 and 2024. A total of 6,438 patients with breast cancer were initially screened. Inclusion criteria were: (i) confirmed invasive breast cancer with HER2 status determined by IHC and/or FISH; (ii) availability of complete three-sequence breast MRI (T1, T2, DCE); and (iii) presence of corresponding clinical and molecular data. Exclusion criteria were: (i) incomplete imaging data; (ii) prior neoadjuvant therapy before imaging; (iii) low-quality MRI scans with artifacts affecting analysis; and (iv) missing or indeterminate HER2 classification.

To enhance transparency while preserving site anonymity, we provide a center-level breakdown of patient enrollment:

- Center A: screened 1,800 → included 671 (HER2+: 360; HER2–: 311); excluded 1,129 (Incomplete MRI: 480; Missing/indeterminate HER2: 370; Low-quality artifacts: 210; Neoadjuvant therapy: 69).
- Center B: screened 1,650 → included 615 (HER2+: 330; HER2–: 285); excluded 1,035 (Incomplete MRI: 430; Missing/indeterminate HER2: 320; Low-quality artifacts: 210; Neoadjuvant therapy: 65; Others 10).
- Center C: screened 1,520 → included 567 (HER2+: 304; HER2–: 263); excluded 953 (Incomplete MRI: 385; Missing/indeterminate HER2: 300; Low-quality artifacts: 200; Neoadjuvant therapy: 68).
- Center D: screened 1,468 → included 547 (HER2+: 292; HER2–: 255); excluded 921 (Incomplete MRI: 360; Missing/indeterminate HER2: 290; Low-quality artifacts: 200; Neoadjuvant therapy: 71).

In total, 2,400 patients were included in the final analysis (HER2-positive: 1,286; HER2-negative: 1,114), while 4,038 patients were excluded. This center-level distribution ensures proportional representation of HER2-positive and HER2-negative cases across institutions, consistent with the study’s inclusion and exclusion criteria (**Supplementary Figure S1**).

To assess potential selection bias, baseline characteristics of excluded patients (n = 6,438) were compared with those of included patients (n = 2,400). Results of this comparison are summarized in **Supplementary Table S3**.

### 2.2 Imaging data acquisition and preprocessing

#### 2.2.1 MRI protocols and sequences

All breast MRI scans were obtained using 3T MRI scanners across the four participating medical centers, following standardized imaging protocols to maintain consistency across institutions. Each patient underwent three essential MRI sequences: T1-weighted (T1), T2-weighted (T2), and T2-weighted with contrast enhancement (T2+contrast). For contrast-enhanced imaging, a gadolinium-based contrast agent was administered at a standardized dose of 0.1 mmol/kg, followed by a 20 mL saline flush to ensure optimal vascular distribution. All imaging was performed in the axial plane, providing high spatial resolution necessary for accurate tumor characterization and feature extraction. Detailed MRI acquisition parameters, including scanner models, voxel sizes, and contrast agent types used at each center, are summarized in **Table 1**.

**Table 1:** MRI acquisition parameters across centers.

Parameter	Center A	Center B	Center C	Center D
MRI Scanner Model	Siemens Prisma	GE Discovery	Philips Ingenia	Siemens Skyra
Voxel Size (mm³)	0.5 × 0.5 × 3	0.6 × 0.6 × 3	0.5 × 0.5 × 3	0.6 × 0.6 × 3
Slice Thickness (mm)	3	3	3	3
In-Plane Resolution	512 × 512	512 × 512	512 × 512	512 × 512

#### 2.2.2 Image preprocessing, standardization, and image segmentation

To ensure consistency and comparability across imaging data, a series of preprocessing steps were applied prior to feature extraction. Intensity normalization was performed to standardize voxel intensity values and mitigate scanner-related variations. To correct for positional discrepancies between sequences, rigid-body registration was applied, aligning images within each patient. Motion-related distortions were addressed through automated artifact detection and correction algorithms, enhancing image quality. Additionally, all MRI scans were resampled to a uniform voxel size of 0.5 × 0.5 × 3 mm³, ensuring consistency across datasets. Finally, images were converted into the NIfTI format, facilitating structured data input for deep learning analysis. These preprocessing steps collectively ensured high-quality, standardized imaging data, minimizing variations arising from differences in acquisition protocols and scanner specifications (11–13).

Image Segmentation was conducted by two experienced radiologists with 14 and 17 years of expertise in breast imaging. Each radiologist independently delineated the tumor regions on MRI scans, ensuring precise segmentation. Discrepancies between the two experts were resolved through consensus review, minimizing interobserver variability. This manual segmentation approach ensured accurate identification of tumor boundaries, providing high-quality input for deep learning-based feature extraction and subsequent analysis (14). To evaluate preprocessing effectiveness, voxel intensity histograms and feature reproducibility metrics were compared before and after harmonization. Intensity normalization and resampling reduced inter-scanner variance, and subsequent ICC filtering ensured retention of stable features (ICC ≥ 0.9).

### 2.3 Deep learning-based feature extraction

#### 2.3.1 Overview of deep learning models

Deep learning models were employed to extract high-dimensional imaging features from breast MRI scans. Unlike traditional radiomics, which relies on manually engineered features, deep learning-based feature extraction enables automatic learning of hierarchical representations, capturing complex spatial and textural patterns. In this study, four state-of-the-art deep learning architectures were utilized for feature extraction: ResNet50, VGG16, EfficientNet-B0, and Vision Transformer (ViT-Small). These models were selected based on their demonstrated effectiveness in medical image analysis and their ability to capture both local and global image features.

#### 2.3.2 Feature extraction using ResNet50, VGG16, EfficientNet-B0, and ViT-Small

Each MRI sequence (T1, T2, and T2+contrast) was processed through the pre-trained deep learning models, which were modified to extract deep features from the final fully connected layers. Feature extraction was restricted to the radiologist-delineated tumor regions of interest (ROIs) rather than entire breast volumes, ensuring that the learned features corresponded to tumor-specific morphology, enhancement kinetics, and peritumoral characteristics. The models were pretrained on ImageNet and then adapted for feature extraction without fine-tuning, ensuring robust feature extraction without the need for additional training on limited medical datasets. Pilot experiments comparing fine-tuned versus frozen-weight strategies indicated that fine-tuning led to overfitting, while frozen-weight feature extraction provided more stable cross-center generalization. Detailed results of this comparison are presented in **Supplementary Table S1**. The extracted feature vectors were high-dimensional, capturing a diverse range of spatial, textural, and structural characteristics from the MRI images.

The feature dimensions extracted from each MRI sequence varied across the deep learning models used in this study. ResNet50 generated a 2048-dimensional feature vector per sequence, while VGG16 produced a higher-dimensional representation with 4096 features per sequence. EfficientNet-B0, known for its efficiency in feature extraction, provided a 1280-dimensional feature set per sequence. In contrast, the Vision Transformer (ViT-Small), leveraging self-attention mechanisms, extracted a 384-dimensional feature vector per sequence. These varying feature dimensions reflect the unique architectures and representation capabilities of each model, influencing their ability to capture spatial and textural characteristics from breast MRI scans.

#### 2.3.3 Intraclass correlation coefficient selection

To ensure the reliability and reproducibility of the extracted deep features, an Intraclass Correlation Coefficient (ICC) analysis was performed. Features with an ICC above 0.9 were considered highly reproducible and retained for further analysis, while those with lower ICC values were excluded. This step was crucial in eliminating non-robust features and enhancing the stability of the predictive model.

#### 2.3.4 LASSO dimensionality reduction

Given the high dimensionality of deep learning-derived features, Least Absolute Shrinkage and Selection Operator (LASSO) regression was applied for feature selection. LASSO is a sparsity-inducing method that reduces collinearity and retains only the most relevant features by enforcing L1 regularization. This step significantly reduced the feature set while preserving the most informative predictors, improving the interpretability and efficiency of the model in distinguishing HER2 expression levels in breast cancer. Beyond statistical relevance, several of the retained features demonstrated strong biological and clinical interpretability. Notably, features corresponding to washout enhancement kinetics, peritumoral edema, irregular tumor margins, and microcalcifications were among the final predictors. This convergence highlights that the LASSO-selected feature set not only optimized predictive performance but also aligned with known mechanistic correlates of HER2-driven tumor biology.

### 2.4 Clinical feature selection and integration

#### 2.4.1 Statistical analysis of clinical features

To identify the most relevant clinical features for HER2 classification, a statistical analysis was performed on the available clinical dataset. Each clinical feature was assessed for its association with HER2 status using univariate analysis, where features with a p-value < 0.05 were considered statistically significant. This threshold ensured that only features with a meaningful relationship to HER2 expression were retained. As a result, three clinical features were selected for integration into the predictive model.

#### 2.4.2 Regression model development and feature scoring

The selected clinical features were incorporated into a regression model alongside the extracted deep learning-based imaging features. A predictive equation was generated based on patient labels, where each patient had a unique equation derived from the model. This approach enabled the calculation of a Deep Score for every individual, representing their likelihood of HER2 positivity or negativity. By integrating both deep learning-based imaging features and statistically significant clinical variables, the model aimed to improve the accuracy and interpretability of HER2 classification in breast cancer.

### 2.5 Nomogram development and validation

#### 2.5.1 Construction of the nomogram

A nomogram was developed to provide an individualized prediction model for HER2 status by integrating deep learning-based imaging features and selected clinical variables. The nomogram was constructed using a multivariate logistic regression model, where each predictive factor was assigned a weighted score based on its contribution to HER2 classification. The final model incorporated the most relevant deep features extracted from MRI scans, along with the three statistically significant clinical features identified in the previous step. The nomogram visually represents the relationship between these predictive variables and the probability of HER2 positivity, allowing for intuitive clinical interpretation.

#### 2.5.2 Calibration and internal validation

To assess the reliability and predictive accuracy of the nomogram, calibration and internal validation were performed. Calibration was evaluated using a calibration curve, comparing the predicted HER2 probabilities with actual HER2 status across different probability thresholds. Model performance was further validated using bootstrapping with 1,000 resamples to minimize overfitting and estimate the model’s generalizability. The Hosmer-Lemeshow goodness-of-fit test was conducted to measure how well the predicted probabilities aligned with observed outcomes. These validation techniques ensured the robustness of the nomogram, enhancing its potential for clinical application in HER2 classification.

### 2.6 Model performance evaluation

The performance of the predictive models was assessed using ROC analysis. The Area Under the Curve (AUC) was used as the primary metric to evaluate the discriminative ability of the models in distinguishing HER2-positive from HER2-negative cases. Higher AUC values indicate better classification performance. ROC curves were generated for each deep learning model (ResNet50, VGG16, EfficientNet-B0, and ViT-Small) as well as for the integrated nomogram. Comparisons were made to determine the most effective approach for HER2 classification (15–17).

To assess the clinical utility and net benefit of the developed models, DCA was performed. DCA evaluates the model’s effectiveness across a range of probability thresholds, providing insight into its potential impact in clinical decision-making. The net benefit was calculated by incorporating both true-positive and false-positive classifications, ensuring that the models provide meaningful risk stratification for HER2 classification. By comparing the DCA curves of individual deep learning models and the final nomogram, the most clinically applicable model was identified.

### 2.7 Statistical analysis

#### 2.7.1 Software and tools used

All statistical analyses and model evaluations were conducted using Python (version 3.9.12) and R (version 4.2.2). Deep learning-based feature extraction was performed using TensorFlow (version 2.10.0) and PyTorch (version 1.13.0). The scikit-learn (version 1.1.1) library was utilized for machine learning and statistical modeling, including LASSO regression and logistic regression analysis. Nomogram construction and validation were carried out using the rms (version 6.4-0) package in R. ROC curve analysis and AUC calculations were performed using the pROC (version 1.18.0) package, while DCA was conducted with the dcurves (version 0.3.1) package.

#### 2.7.2 Statistical tests and significance criteria

Descriptive statistics were used to summarize patient characteristics. Chi-square tests and Fisher’s exact tests were applied to compare categorical variables, while independent t-tests and Mann-Whitney U tests were used for continuous variables. The ICC was calculated to assess feature reproducibility, with an ICC threshold of 0.9 for feature selection. LASSO regression was employed for dimensionality reduction, and multivariate logistic regression was used to construct the predictive model. Calibration of the nomogram was assessed using the Hosmer-Lemeshow goodness-of-fit test, and statistical significance was defined as p < 0.05 for all analyses.

## 3 Results

### 3.1 Patient characteristics and clinical feature analysis

**Figure 1:**
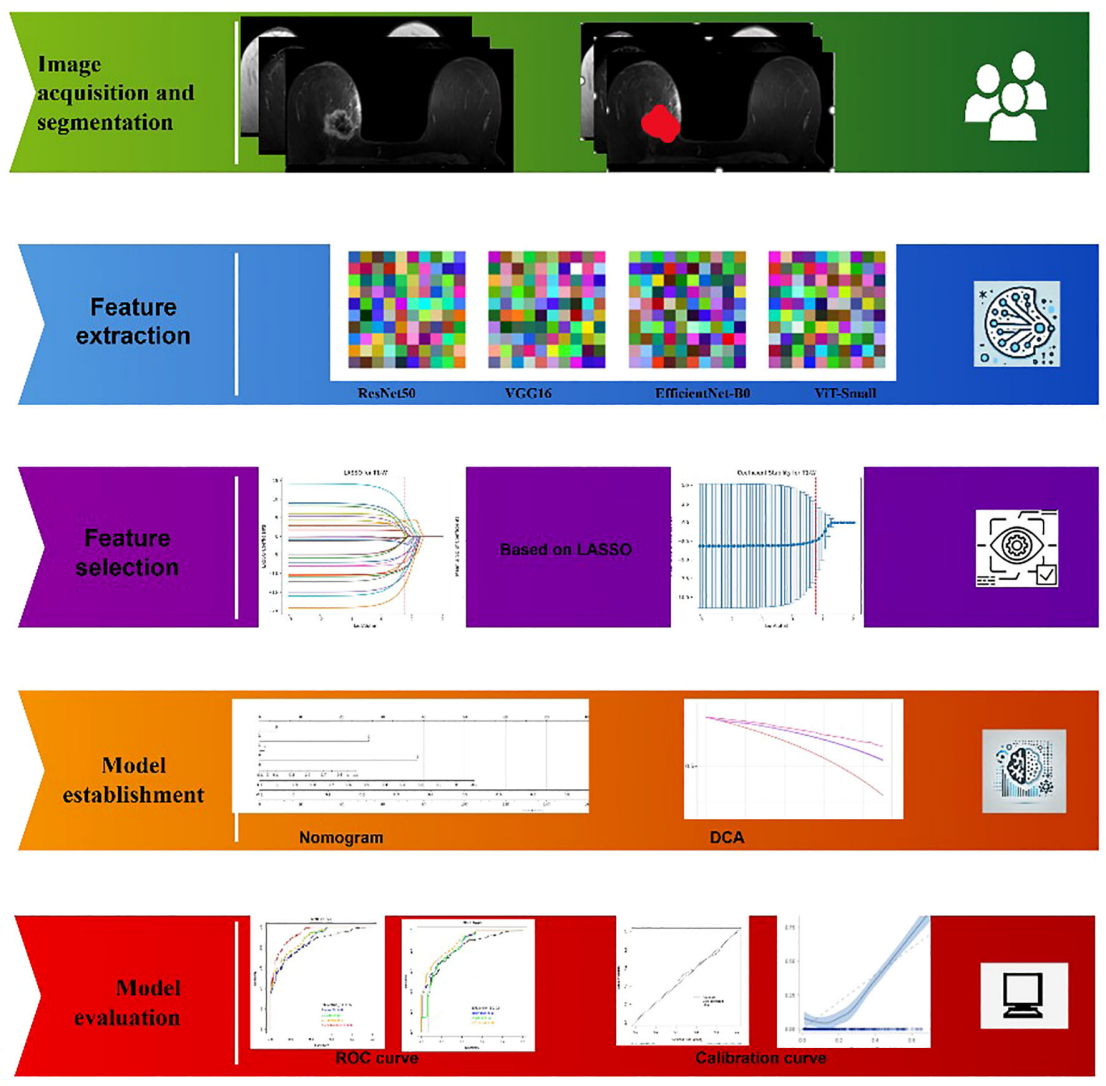
Overview of the proposed framework.

Based on a retrospective study, which provides a detailed comparison of demographic, clinicopathological, molecular, transcriptomic, and proteomic characteristics between HER2-positive (n = 1,286) and HER2-negative (n = 1,114) breast cancer patients (**Figure 1**). Baseline characteristics across the four centers were compared and found to be well-balanced (**Supplementary Table S2**). No significant inter-center differences were observed for age, menopausal status, tumor size, histological type, or molecular subtype (all p > 0.05). In addition, supplementary analysis confirmed that preprocessing (intensity normalization, resampling, registration) effectively reduced inter-scanner variability, as demonstrated by improved consistency in voxel intensity distributions and feature reproducibility metrics.

Baseline characteristics of excluded patients were similar to those of the final cohort, with no significant differences in age, menopausal status, or histological subtype. Tumor size was slightly smaller among excluded patients (2.4 cm vs. 2.6 cm, p = 0.08), but this was not clinically significant. These findings suggest minimal risk of selection bias (**Supplementary Table S3**).

#### 3.1.1 Demographic and clinical data distribution

The mean age of patients with HER2-positive tumors was 54.7 ± 10.9 years, while HER2-negative patients had a mean age of 55.1 ± 11.2 years, showing no statistically significant difference (p = 0.719). Similarly, the menopausal status distribution was comparable between both groups (p = 0.165). Tumor laterality (left vs. right breast) was evenly distributed, with no significant association with HER2 status (p = 0.372). The mean tumor size was slightly larger in HER2-positive cases (2.7 ± 1.1 cm) compared to HER2-negative tumors (2.5 ± 1.0 cm), but the difference was not statistically significant (p = 0.642). The predominant histological subtype in both groups was invasive ductal carcinoma (92.5% in HER2-positive vs. 90.7% in HER2-negative, p = 0.142), and lymph node involvement rates were comparable (p = 0.294). HER2-positive tumors were more frequently classified as Grade 3 (poorly differentiated) (33.1% vs. 20.1%), although this difference was not statistically significant (p = 0.362) (**Table 2**).

**Table 2:** Demographic characteristics of patients.

Feature	HER2-positive (n = 1286)	HER2-negative (n = 1114)	P value
Age (years, mean ± SD)	54.7 ± 10.9	55.1 ± 11.2	0.719
Menopausal Status			0.165
- Postmenopausal	820 (63.8%)	720 (64.6%)	
- Premenopausal	466 (36.2%)	394 (35.4%)	
Tumor Location			0.372
- Left Breast	670 (52.1%)	590 (53.0%)	
- Right Breast	616 (47.9%)	524 (47.0%)	
Tumor Size (cm, mean ± SD)	2.7 ± 1.1	2.5 ± 1.0	0.642
Histological Type			0.142
- Invasive Ductal Carcinoma	1190 (92.5%)	1010 (90.7%)	
- Other Types	96 (7.5%)	104 (9.3%)	
Hormone Receptor Status			0.891
- ER-Positive	900 (70.0%)	890 (79.9%)	
- ER-Negative	386 (30.0%)	224 (20.1%)	
- PR-Positive	860 (66.9%)	780 (70.0%)	0.538
- PR-Negative	426 (33.1%)	334 (30.0%)	
Lymph Node Involvement			0.294
- Positive	640 (49.8%)	520 (46.7%)	
- Negative	646 (50.2%)	594 (53.3%)	
Molecular Subtype			0.189
- Luminal B (HER2+)	460 (35.8%)	–	
- HER2-Enriched	826 (64.2%)	–	
- Luminal A	–	640 (57.4%)	
- Luminal B (HER2-)	–	334 (30.0%)	
- Triple-Negative	–	140 (12.6%)	
Tumor Grade			0.362
- Grade 1 (Well Differentiated)	310 (24.1%)	480 (43.1%)	
- Grade 2 (Moderately Differentiated)	550 (42.8%)	410 (36.8%)	
- Grade 3 (Poorly Differentiated)	426 (33.1%)	224 (20.1%)	
Genomic Alterations			0.003
- PIK3CA Mutation	700 (54.4%)	340 (30.5%)	
- TP53 Mutation	460 (35.8%)	270 (24.2%)	
- ERBB2 Amplification	1180 (91.8%)	64 (5.7%)	
- BRCA1/BRCA2 Mutation	280 (21.8%)	210 (18.9%)	
Transcriptomic Features			0.006
- ESR1 (Estrogen Receptor)	High in 860 (66.9%)	High in 890 (79.9%)	
- HER2 (ERBB2) mRNA Level	High in 1286 (100%)	Low in 1114 (100%)	
Proteomic Features			0.015
- HER2 Protein Expression	High in 1286 (100%)	Low in 1114 (100%)	
- P53 Protein Expression	High in 460 (35.8%)	High in 270 (24.2%)	

#### 3.1.2 Comparison of selected clinical features between HER2-positive and HER2-negative groups

In terms of hormone receptor status, estrogen receptor (ER)-negative tumors were more common among HER2-positive cases (30.0% vs. 20.1%), whereas progesterone receptor (PR) expression showed no significant variation between the groups. Regarding molecular subtypes, HER2-positive tumors were classified into Luminal B (HER2+) (35.8%) and HER2-Enriched (64.2%), while HER2-negative cases included Luminal A (57.4%), Luminal B (HER2-) (30.0%), and Triple-Negative (12.6%) subtypes.

Genomic alterations were significantly different between HER2-positive and HER2-negative groups. PIK3CA mutations were more frequent in HER2-positive tumors (54.4% vs. 30.5%, p = 0.003), as were TP53 mutations (35.8% vs. 24.2%). The hallmark feature of HER2-positive cases was ERBB2 amplification, detected in 91.8% of HER2-positive tumors compared to only 5.7% of HER2-negative cases. BRCA1/BRCA2 mutation rates were slightly higher in HER2-positive cases, but this difference was not statistically significant. At the transcriptomic and proteomic levels, HER2 (ERBB2) mRNA expression was elevated in 100% of HER2-positive tumors, while it was low in 100% of HER2-negative cases (p = 0.006). Similarly, HER2 protein expression was consistently higher in HER2-positive tumors but low in HER2-negative tumors (p = 0.015). Additionally, P53 protein expression was observed more frequently in HER2-positive cases (35.8% vs. 24.2%), further distinguishing the two groups.

#### 3.1.3 Imaging and tumor characteristics in HER2-positive vs. HER2-negative breast cancer

The imaging characteristics of HER2-positive and HER2-negative breast cancers exhibited distinct patterns across various modalities, as summarized in **Table 3**. HER2-positive tumors were significantly larger than HER2-negative tumors, with a mean size of 2.7 ± 1.1 cm vs. 2.5 ± 1.0 cm, respectively (p = 0.03). Although single tumors were more frequently observed in HER2-negative cases (63.7% vs. 52.1% in HER2-positive cases), this difference was not statistically significant (p = 0.07). Axillary lymph node involvement was notably higher in HER2-positive tumors (63.0% vs. 44.0%, p < 0.001), and these tumors more frequently exhibited cortical thickness ≥3 mm (48.2% vs. 35.0%, p = 0.06), suggesting a greater likelihood of nodal metastasis.

**Table 3:** Imaging characteristics of patients.

Feature	HER2-positive (n = 1286)	HER2-negative (n = 1114)	P value
Tumor Size (cm)	Mean 2.7 ± 1.1	Mean 2.5 ± 1.0	0.03
Number of Tumors	Single in 670 (52.1%)	Single in 710 (63.7%)	0.07
Abnormal Axillary Lymph Nodes	Present in 810 (63.0%)	Present in 490 (44.0%)	<0.001
Cortical Thickness of Axillary Lymph Nodes	≥3mm in 620 (48.2%)	≥3mm in 390 (35.0%)	0.06
Presence of Microcalcifications	Present in 780 (60.7%)	Present in 470 (42.2%)	0.002
Posterior Acoustic Features on Ultrasound	Shadowing in 730 (56.8%)	Shadowing in 600 (53.9%)	0.10
Tumor Margin on Ultrasound	Irregular in 1060 (82.5%)	Irregular in 850 (76.3%)	0.04
Tumor Shape on MRI	Irregular in 1080 (84.0%)	Irregular in 900 (80.8%)	0.09
MRI Enhancement Pattern	Heterogeneous in 950 (73.9%)	Heterogeneous in 910 (81.7%)	0.06
Enhancement Curve on MRI	Washout in 520 (40.4%)	Washout in 310 (27.8%)	0.003
Nonmass Enhancement	Present in 410 (31.9%)	Present in 320 (28.7%)	0.08
Distribution of Nonmass Enhancement	Segmental in 330 (25.7%)	Segmental in 290 (26.0%)	0.95

Microcalcifications were significantly more common in HER2-positive tumors (60.7% vs. 42.2%, p = 0.002), highlighting their potential role as an imaging biomarker for HER2 classification. On ultrasound imaging, HER2-positive tumors more frequently displayed irregular margins (82.5% vs. 76.3%, p = 0.04), indicative of a more aggressive growth pattern, while posterior acoustic shadowing was observed at similar rates in both groups (p = 0.10). MRI characteristics also revealed key differences between HER2 subtypes. HER2-positive tumors demonstrated a slightly higher prevalence of irregular shapes (84.0% vs. 80.8%, p = 0.09) and a washout enhancement curve, which was significantly more frequent (40.4% vs. 27.8%, p = 0.003), suggesting more aggressive contrast uptake and rapid contrast clearance. Heterogeneous enhancement was present in 73.9% of HER2-positive cases compared to 81.7% of HER2-negative cases (p = 0.06), while nonmass enhancement (NME) frequency was comparable between groups (31.9% vs. 28.7%, p = 0.08). Moreover, peritumoral edema, a marker associated with increased tumor invasiveness, was significantly more common in HER2-positive tumors (77.0% vs. 65.5%, p = 0.001).

These findings suggest that tumor size, axillary lymph node involvement, microcalcifications, and specific MRI enhancement patterns may serve as key imaging biomarkers for HER2 classification. The higher prevalence of washout enhancement, peritumoral edema, and irregular tumor margins in HER2-positive tumors aligns with their more aggressive biological behavior. The integration of these imaging features with molecular, transcriptomic, and proteomic profiles may enhance precision oncology strategies, aiding in targeted therapy selection and prognostic assessment.

### 3.2 Deep learning feature analysis

#### 3.2.1 ICC-based feature selection results

**Figure 2:**
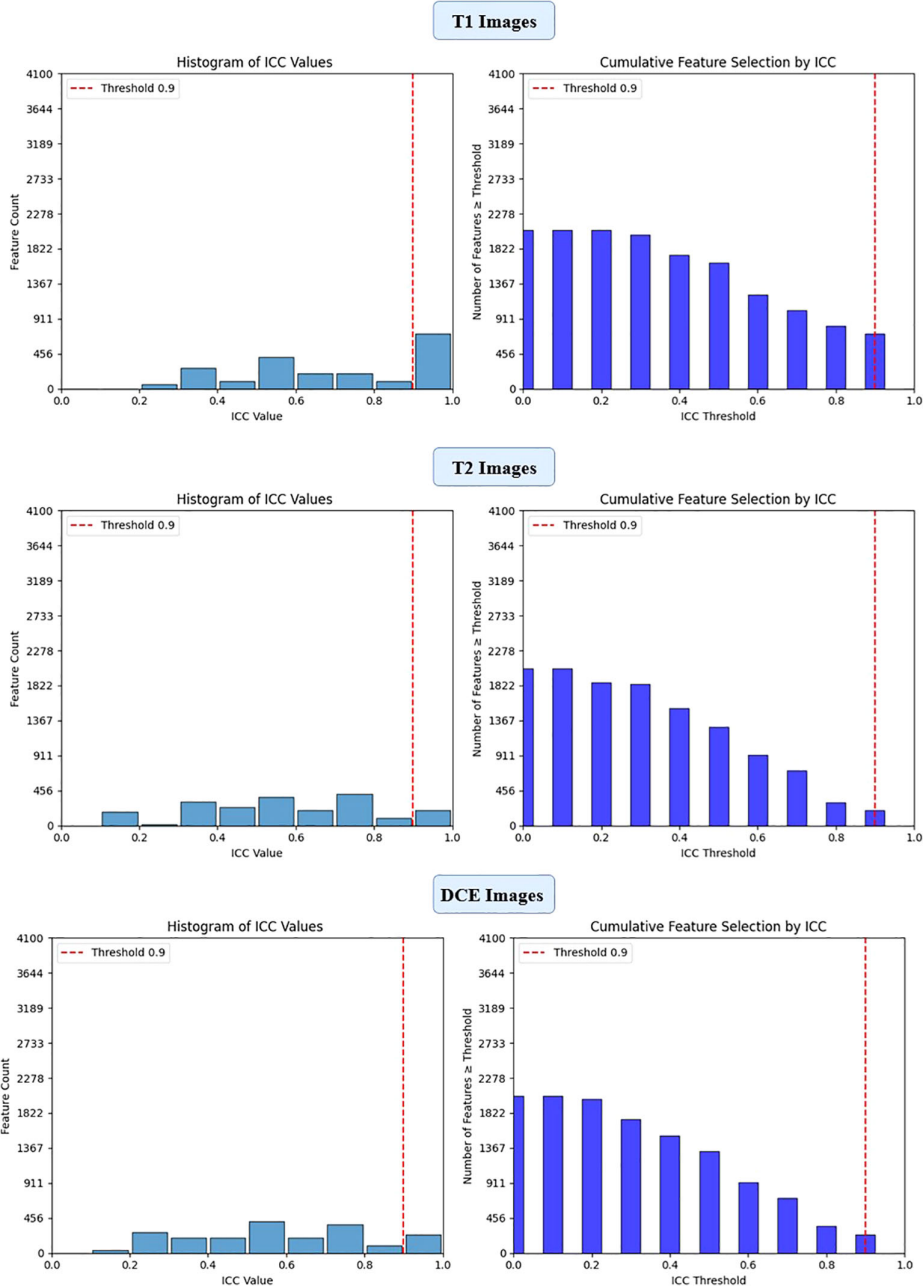
ICC filtering process for ResNet50 model - proportion of retained features across T1-W, T2-W, and DCE sequences.

**Figure 3:**
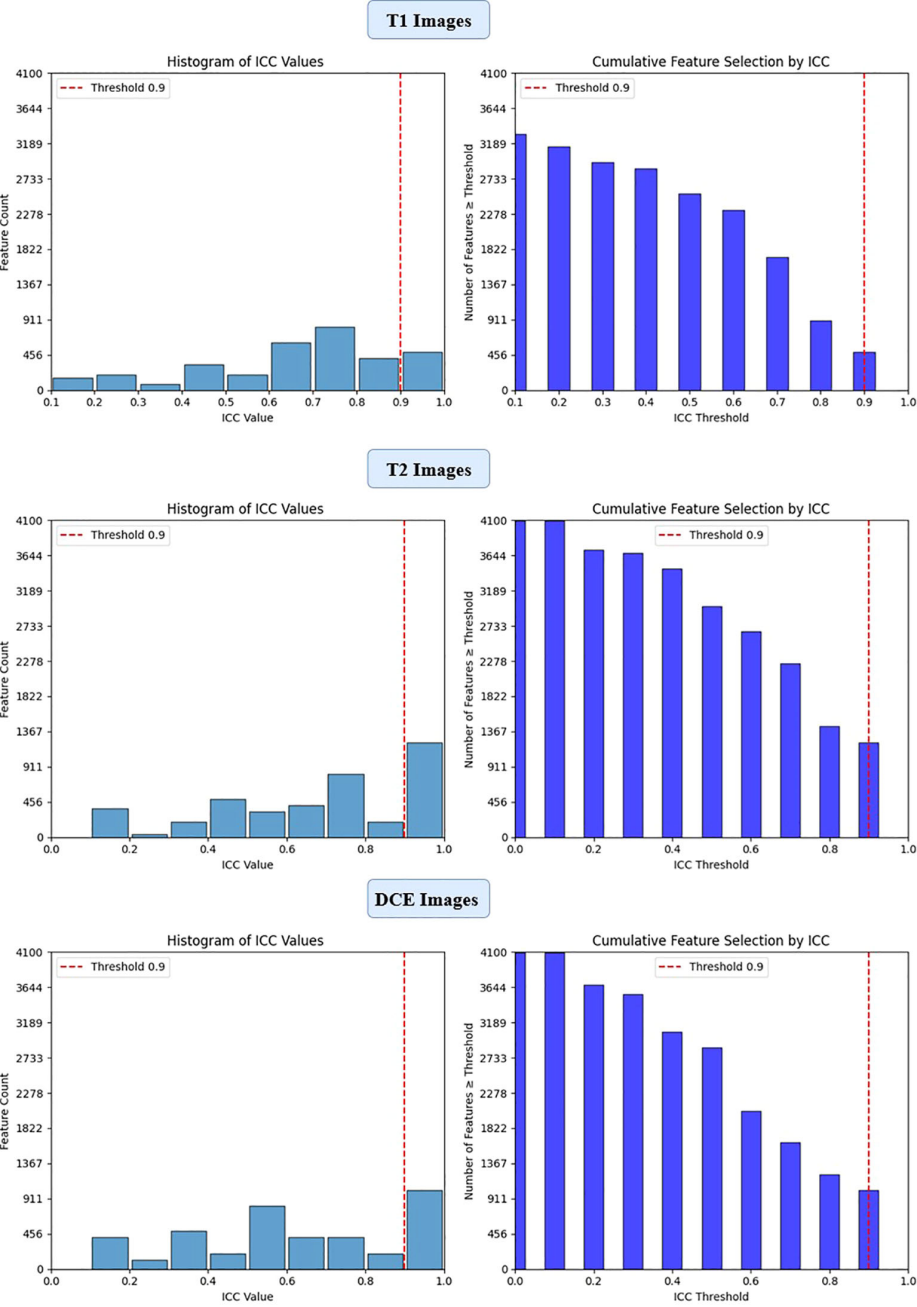
ICC filtering process for VGG16 model - proportion of retained features across T1-W, T2-W, and DCE sequences.

**Figure 4:**
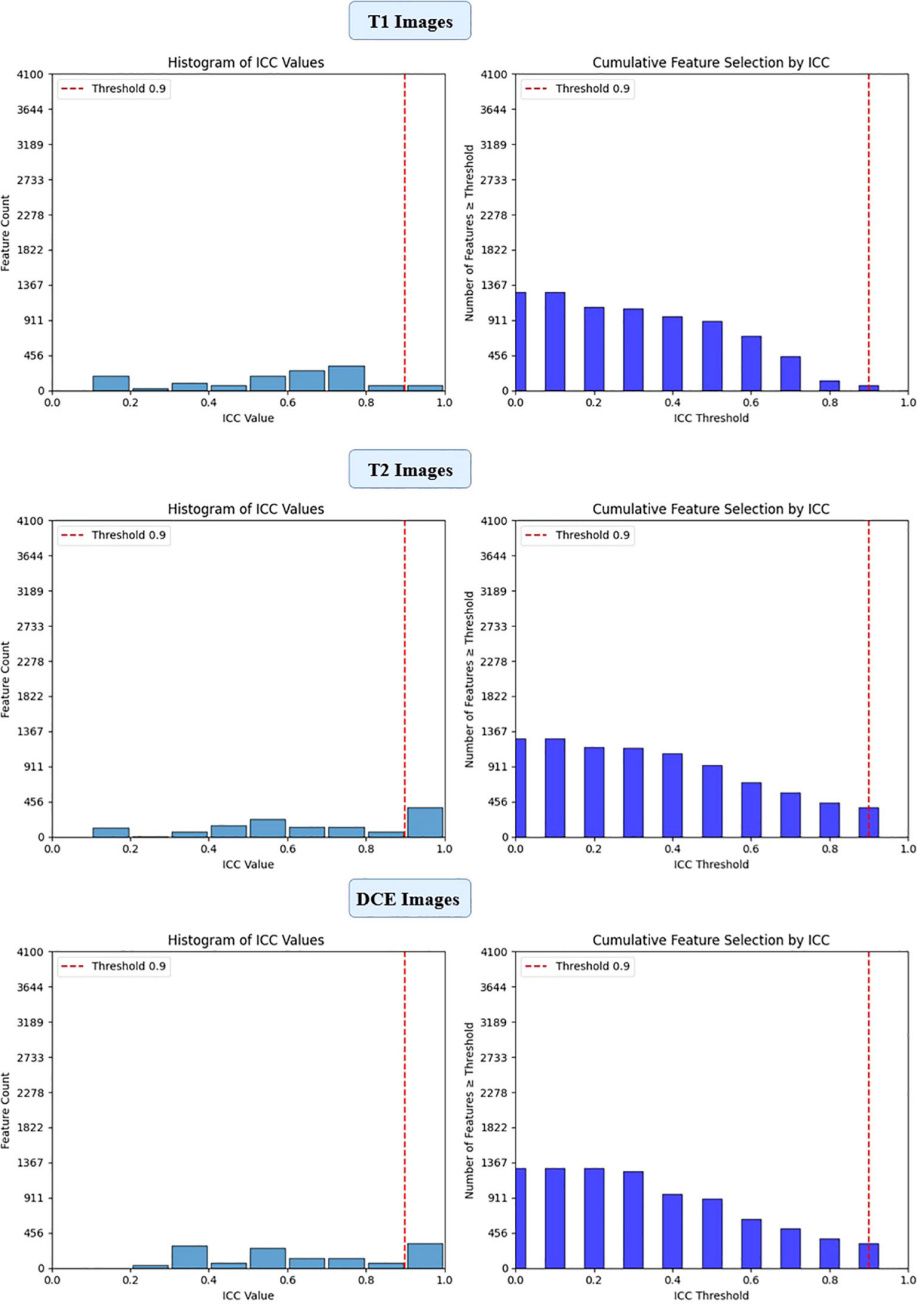
ICC filtering process for efficientNet-B0 model - proportion of retained features across T1-W, T2-W, and DCE sequences.

To ensure the reliability of extracted imaging features, Intraclass Correlation Coefficient (ICC) analysis was performed for each deep learning model across the three MRI sequences (T1-weighted, T2-weighted, and contrast-enhanced (DCE) MRI). Features with ICC ≥ 0.9 were retained, ensuring high reproducibility. The number of selected features varied across models, reflecting differences in their representational capacity and sensitivity to imaging sequences. VGG16 preserved the highest number of features, with 819, 1228, and 1024 features retained from T1-W, T2-W, and DCE, respectively. In contrast, ViT-Small, which uses an attention-based approach, retained the lowest number of features, with 96, 116, and 134 features extracted from the respective sequences. ResNet50 demonstrated a balanced extraction pattern, preserving 308, 204, and 246 features, while EfficientNet-B0 exhibited a more selective feature extraction process, particularly in T2-W imaging, where it retained 384 features, significantly more than in T1-W (64 features). These results highlight VGG16’s capacity for high-dimensional feature extraction, while ViT-Small’s selective attention mechanism leads to a more compact representation. **Figure 2**, **3**, **4**, **5** illustrates the ICC filtering process, showing the proportion of retained features for each model and MRI sequence.

**Figure 5:**
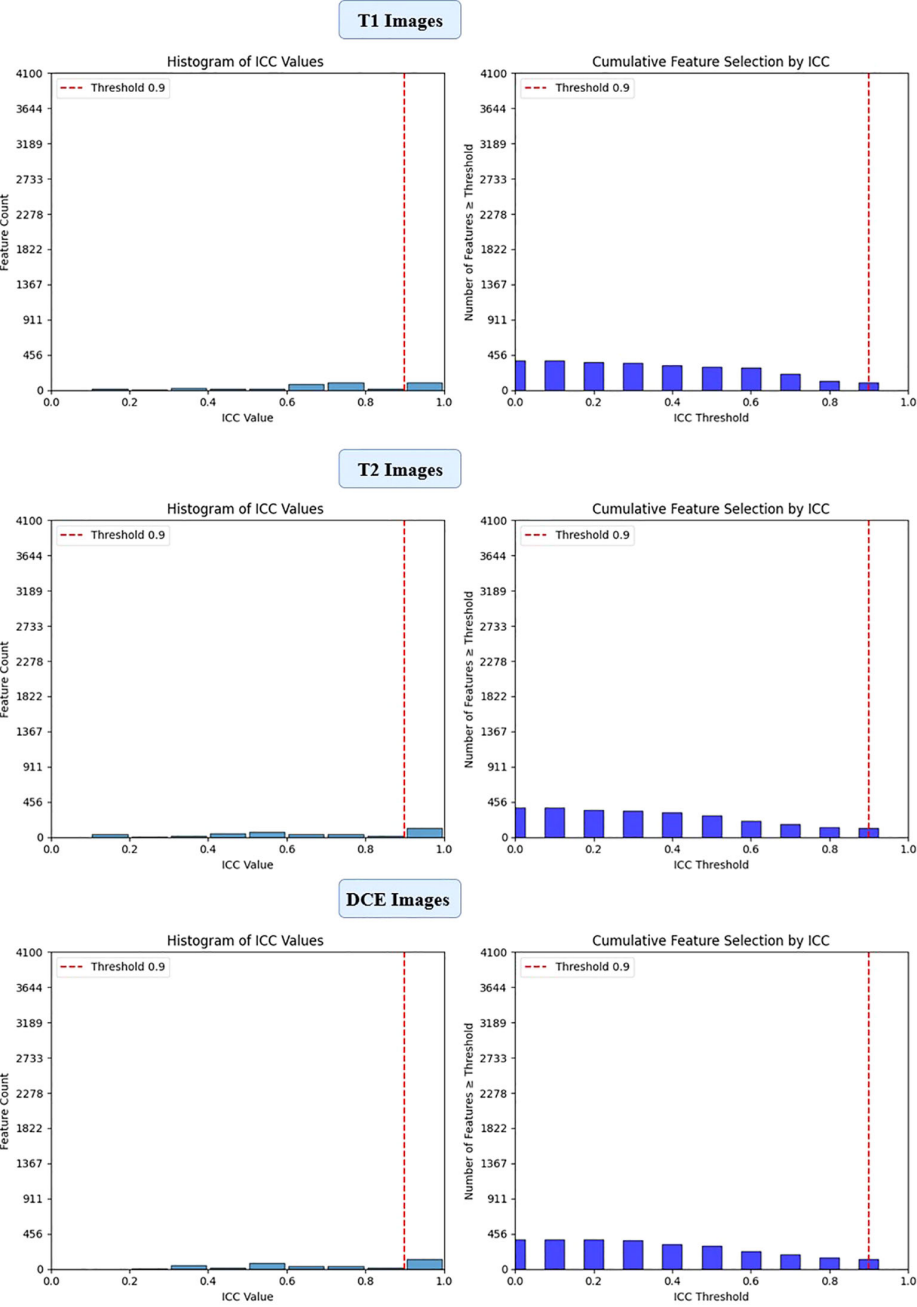
ICC filtering process for ViT-small model - proportion of retained features across T1-W, T2-W, and DCE sequences.

#### 3.2.2 Analysis of feature selection using LASSO

The feature selection process using LASSO regression is a critical step to ensure that only the most relevant and reproducible features are retained for further analysis. In this study, LASSO was applied to the features extracted from four deep learning models (ResNet50, VGG16, EfficientNet-B0, and ViT-Small) across three MRI sequences (T1-weighted, T2-weighted, and DCE) to identify the most significant features for predicting HER2 status in breast cancer.

**Figure 6:**
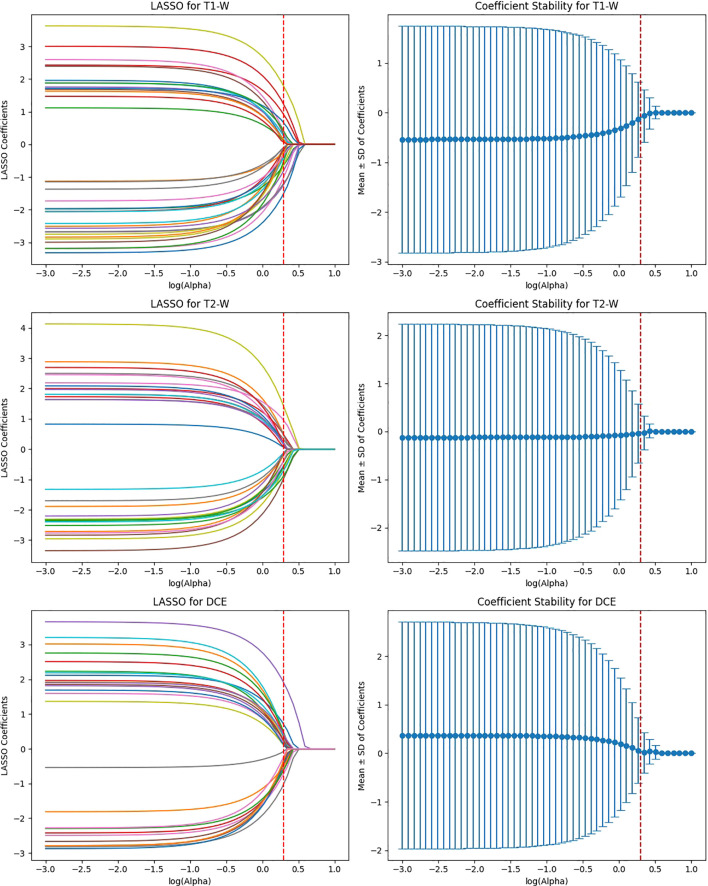
Feature selection using LASSO regression on deep features extracted from ResNet50 model across T1-W, T2-W, and DCE sequences.

**Figure 7:**
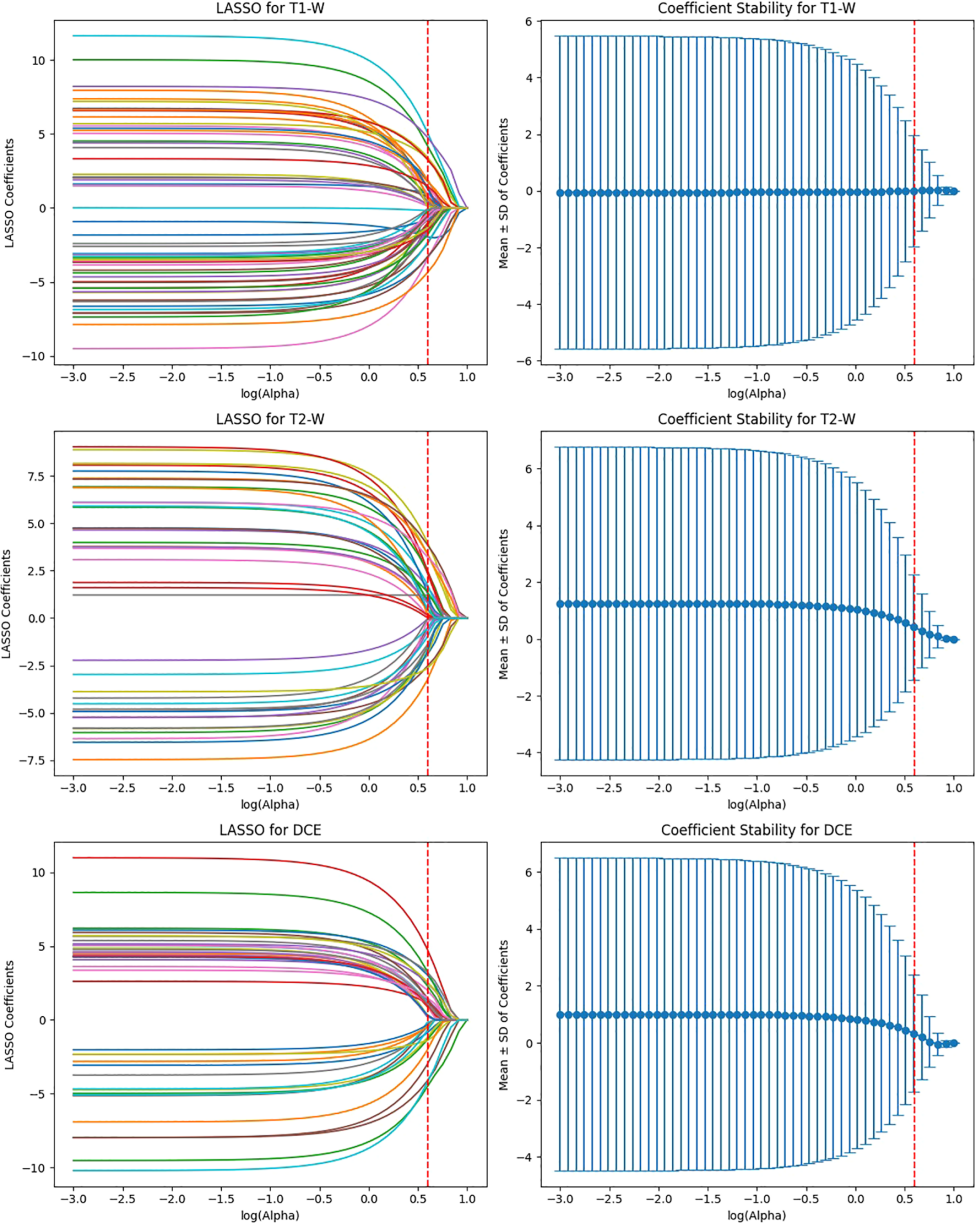
Feature selection using LASSO regression on deep features extracted from VGG16 model across T1-W, T2-W, and DCE sequences.

After the initial ICC filtering, 758 features were retained. The application of LASSO regression resulted in 56 final features. These selected features indicate that, while ResNet50 initially captured a large number of features (6,144), LASSO successfully reduced the feature set, focusing on the most predictive features (**Figure 6**). This indicates that the ResNet50 model has a broad range of features but requires further refinement through LASSO to improve specificity and avoid overfitting. VGG16 extracted the highest number of features (12,288), and after ICC filtering, 3,071 features remained. However, after LASSO optimization, only 124 features were retained. This suggests that VGG16, although capable of extracting a large feature space, still benefits from LASSO’s ability to select a subset of the most relevant features. Despite the large initial feature set, LASSO significantly reduced the complexity, highlighting the power of VGG16 in capturing complex patterns that can be distilled down to a smaller, more manageable set for predictive modeling (**Figure 7**).

**Figure 8:**
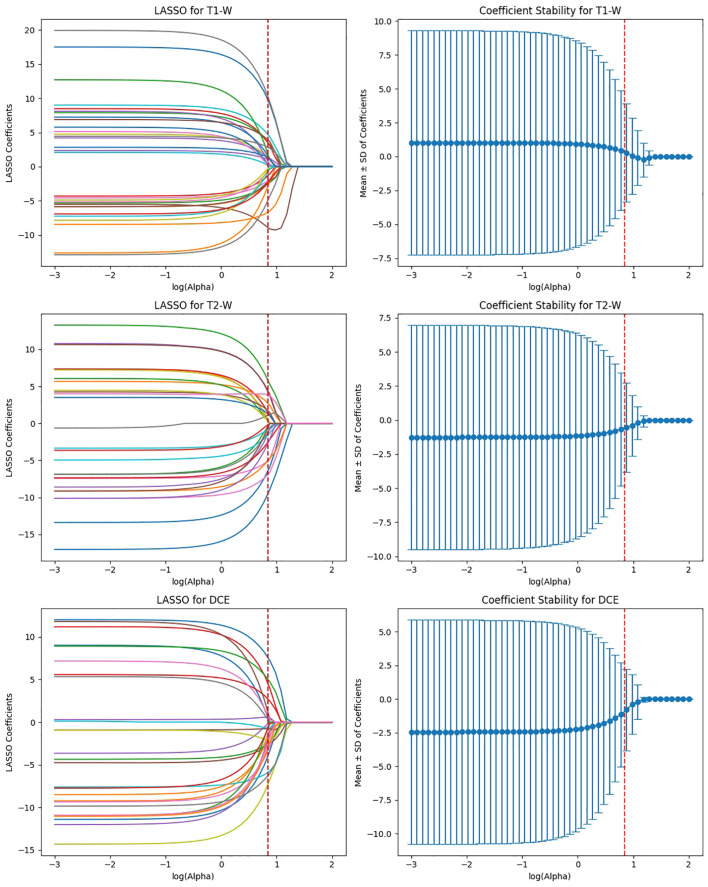
Feature selection using LASSO regression on deep features extracted from efficientNet-B0 model across T1-W, T2-W, and DCE sequences.

EfficientNet-B0 extracted 3,840 features, and after ICC filtering, 768 features remained. Post-LASSO selection, 80 features were retained. While EfficientNet-B0 captured fewer features overall, the final feature set after LASSO was still significant, indicating that the model focuses on a relatively smaller subset of highly predictive features. The ability to reduce feature dimensionality efficiently with LASSO underscores EfficientNet-B0’s effectiveness in feature selection, even with a lower feature count compared to other models (**Figure 8**).

**Figure 9:**
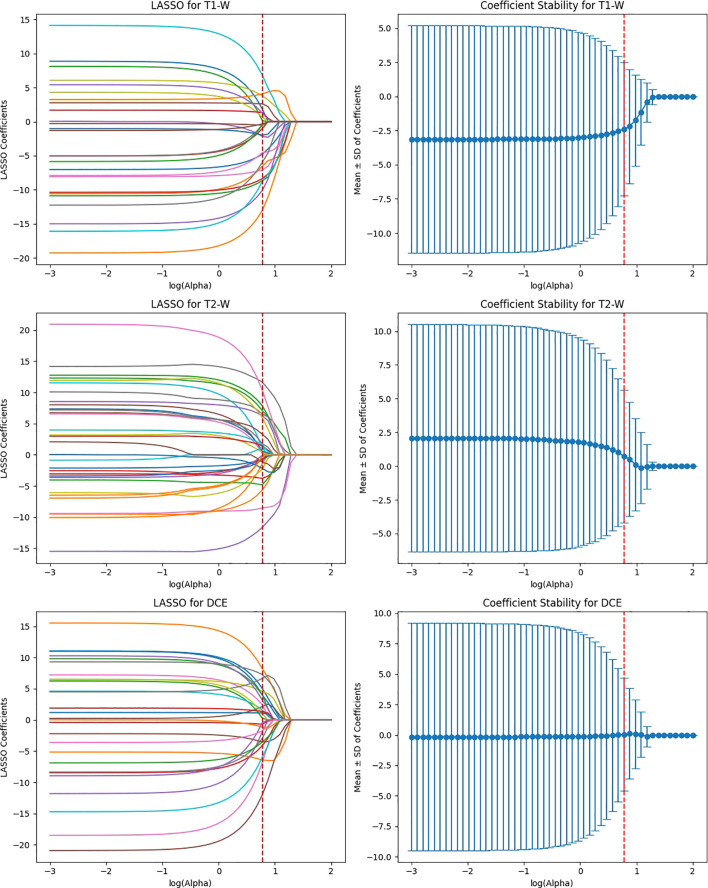
Feature selection using LASSO regression on deep features extracted from ViT-small model across T1-W, T2-W, and DCE sequences.

**Figure 10:**
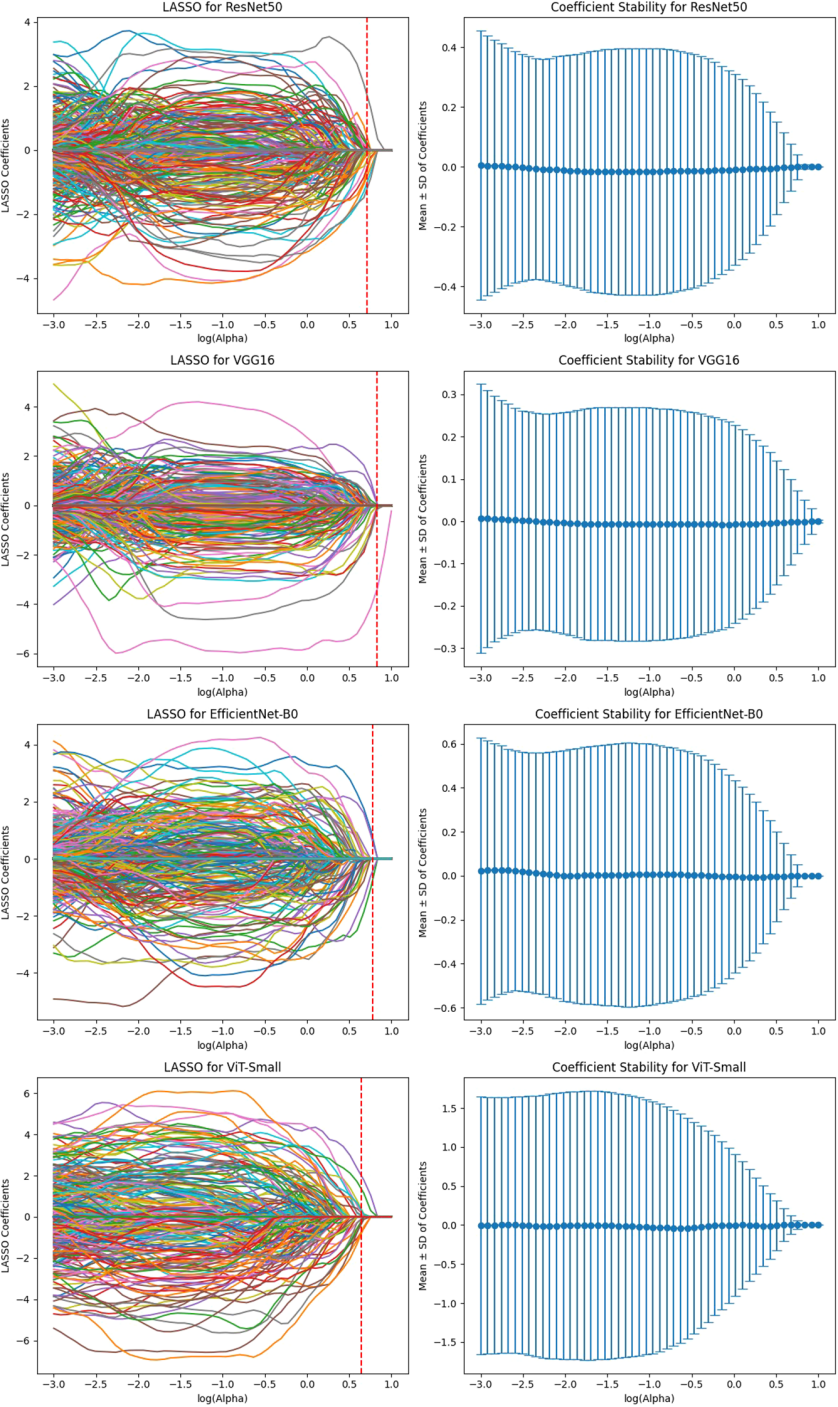
Feature selection using LASSO regression on deep features extracted from ViT-small model across total features (for 3 MRI sequences).

ViT-Small, with the lowest total feature count (1,152), had 346 features after ICC filtering, and only 42 features were selected after LASSO optimization. The low number of features retained in ViT-Small further demonstrates its more focused and sparse feature representation compared to the other models. This may indicate that ViT-Small, while less comprehensive in its feature extraction, is highly efficient in identifying the most relevant features for the task at hand, ensuring minimal redundancy and overfitting. The LASSO regression step is crucial for refining the model’s feature space, ensuring that the final feature sets consist only of the most informative features for the classification task. The process not only reduces dimensionality but also enhances model interpretability, focusing on the most relevant features while eliminating noise. The differences in feature selection results across the models (with VGG16 retaining the most features and ViT-Small retaining the least) highlight the varying capabilities and sensitivities of each model to the imaging data (**Figure 9**). Overall, the LASSO-selected features provide a more robust foundation for the subsequent model development, reducing complexity and increasing the focus on features that are most likely to contribute to accurate predictions of HER2 status in breast cancer on Deep Features Extracted from ViT-Small Model Across Total Features (**Figure 10**).

**Figure 11:**
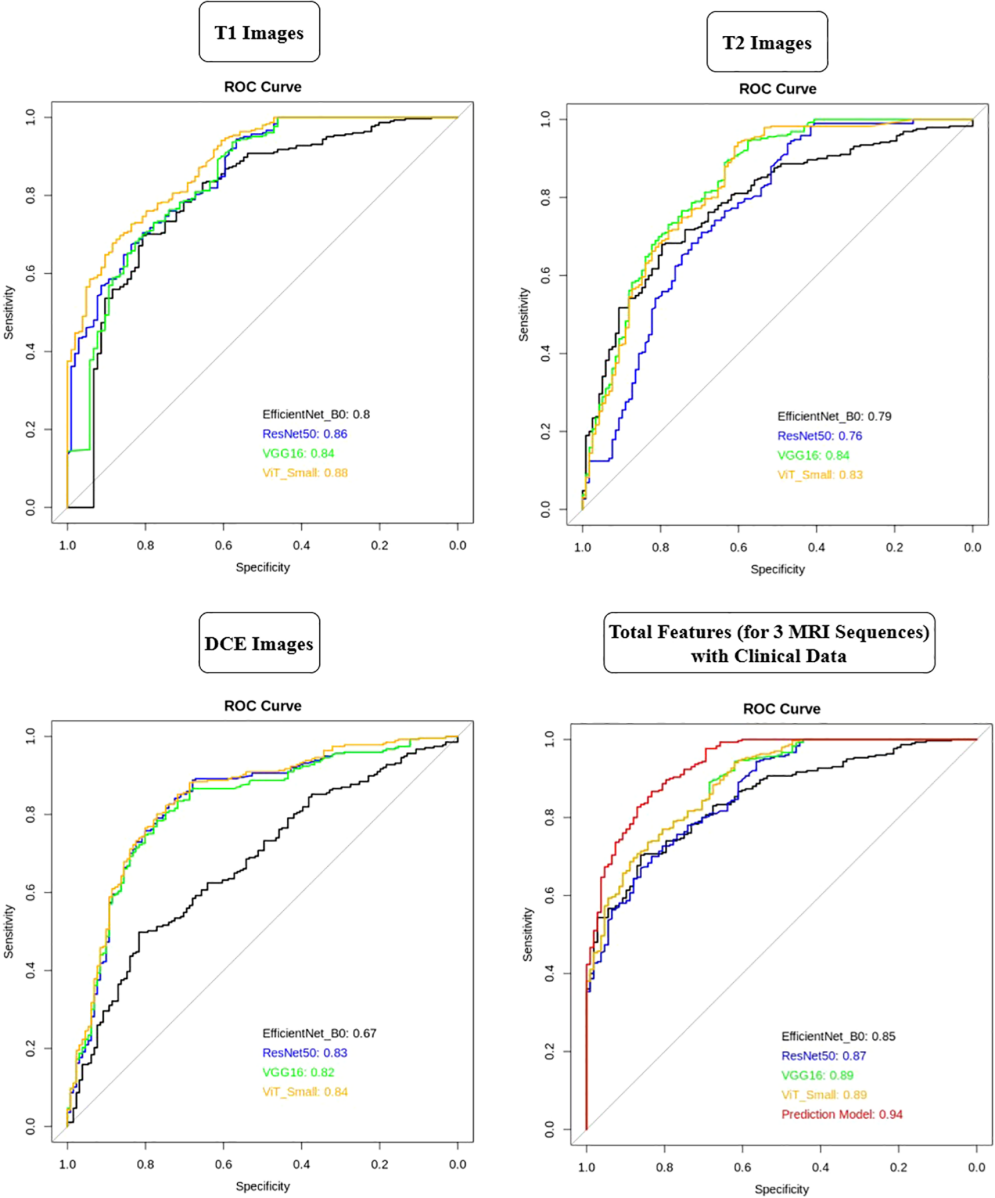
AUC comparison of deep learning models across T1, T2, and DCE MRI sequences with and without clinical data integration.

The AUC analysis reveals that the deep learning models performed differently across various MRI sequences (**Figure 11**). For T1-weighted images, ViT-Small demonstrated the best performance with an AUC of 0.88, followed by ResNet50 and VGG16, with AUCs of 0.86 and 0.84, respectively. EfficientNet-B0 had the lowest AUC of 0.80 for T1-weighted images. In the case of T2-weighted images, VGG16 and ViT-Small showed similar high performance with AUCs of 0.84 and 0.83, respectively, while ResNet50 performed slightly worse at 0.76. For DCE-enhanced images, ViT-Small again outperformed other models with an AUC of 0.84, closely followed by ResNet50 and VGG16, with AUCs of 0.83 and 0.82, respectively. EfficientNet-B0 had a notably lower AUC of 0.67 in this sequence. When integrating all three MRI sequences with clinical data, the models’ performance improved significantly, with VGG16 and ViT-Small achieving an AUC of 0.89, while ResNet50 scored 0.87. EfficientNet-B0 still performed well, achieving an AUC of 0.85, but the Prediction Model, combining all features from the deep learning models and clinical data, achieved the highest AUC of 0.94, illustrating the significant benefit of adding clinical data to improve predictive accuracy.

#### 3.2.3 Comparison of fine-tuned vs. non-fine-tuned models

To evaluate the impact of fine-tuning, we performed a pilot comparison of fine-tuned and frozen-weight feature extraction strategies. Fine-tuning improved training accuracy but resulted in reduced external validation performance, including a drop in cross-center AUCs and poorer calibration. In contrast, frozen-weight feature extraction consistently achieved higher generalization performance. A detailed breakdown of these results is provided in **Supplementary Table S1**.

### 3.3 Nomogram construction and predictive score distribution

**Figure 12:**
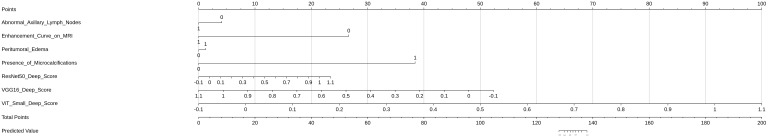
Nomogram for HER2 prediction based on MRI sequences and clinical features.

A nomogram was constructed to provide an individualized risk assessment for HER2-positive breast cancer classification by integrating the selected deep learning-based features from the MRI sequences (T1-weighted, T2-weighted, and DCE) and clinically relevant features (**Figure 12**). The nomogram utilized the ResNet50, VGG16, and ViT-Small deep scores, along with clinical variables such as Abnormal Axillary Lymph Nodes, Enhancement Curve on MRI, Peritumoral Edema, and Presence of Microcalcifications, to generate a comprehensive prediction model for HER2 status.

Each feature in the nomogram was assigned a point value, based on its weight in the final prediction model. The total points were calculated by summing the individual points from each feature, with a higher total score correlating with a greater likelihood of HER2-positive status. The predictive score distribution, as shown in the nomogram, allows clinicians to easily assess an individual patient’s risk of HER2 positivity based on the selected imaging and clinical factors.

#### 3.3.1 Calibration curve analysis

**Figure 13:**
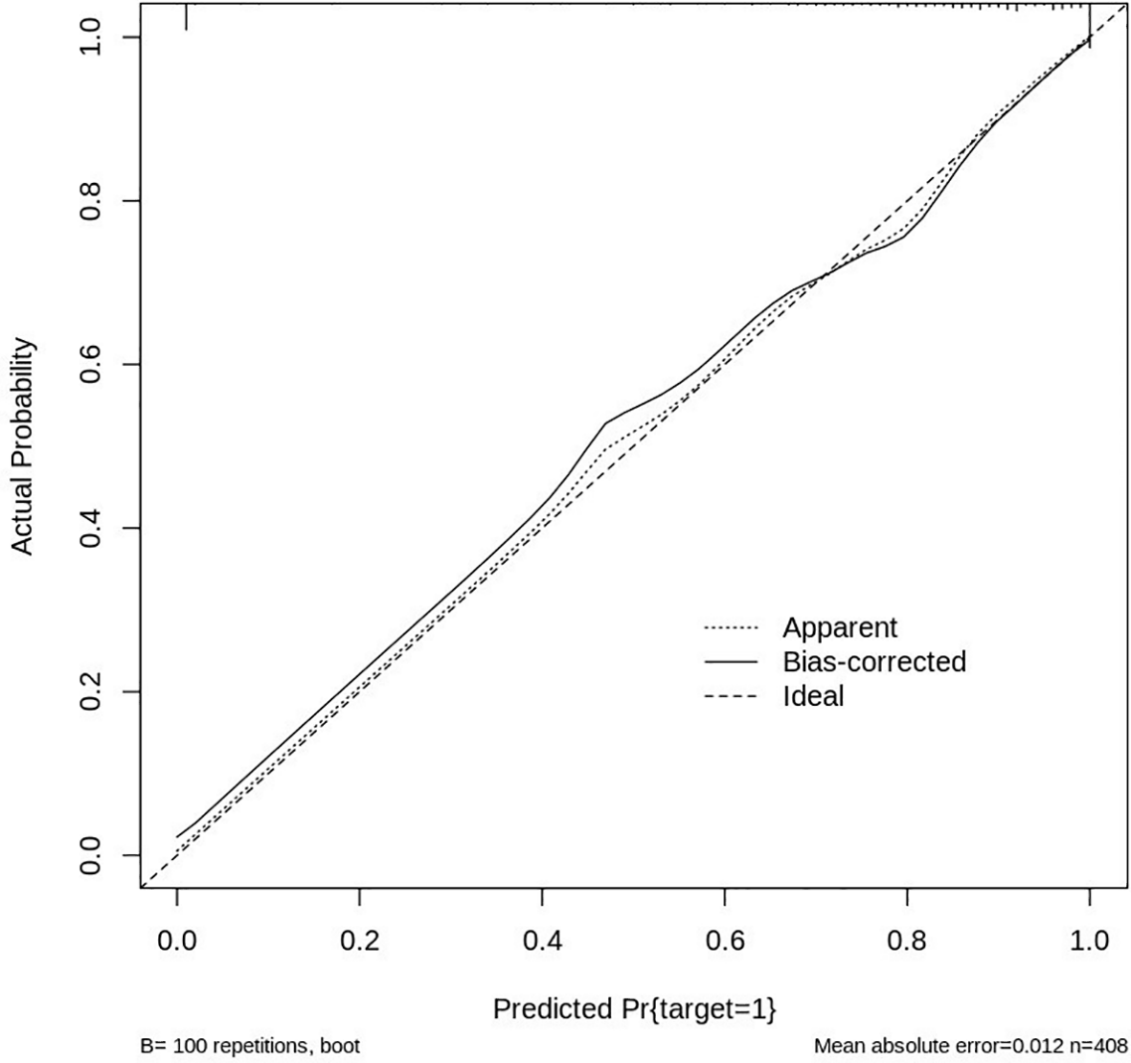
Calibration curve for HER2 prediction model.

The performance and reliability of the nomogram were assessed using a calibration curve, which compares the predicted probabilities of HER2 positivity with the actual observed outcomes. The calibration curve was generated by plotting the predicted HER2 probability against the observed proportion of HER2-positive cases across different thresholds of predicted values. A perfectly calibrated model would produce a 1:1 diagonal line, indicating that the predicted values match the actual outcomes (**Figure 13**).

In this study, the calibration curve demonstrated good agreement between predicted and observed values, with the model showing a strong correlation in predicting HER2 positivity. The Hosmer-Lemeshow test further confirmed the model’s goodness of fit, with no significant deviations from expected values, indicating that the nomogram’s predictions were accurate across the range of risk scores. Overall, the nomogram, integrated with deep learning features and clinical data, proved to be a reliable and effective tool for predicting HER2 status, with good predictive accuracy and strong calibration, making it a valuable asset for personalized treatment planning in breast cancer management.

### 3.4 Model performance evaluation

The DCA was performed to assess the clinical utility of the predictive models developed in this study, including both imaging features from deep learning models and clinical variables. The DCA evaluates the net benefit of each model across different threshold probabilities by comparing the true positive rate and the false positive rate, helping to identify the optimal threshold for clinical decision-making.

**Figure 14:**
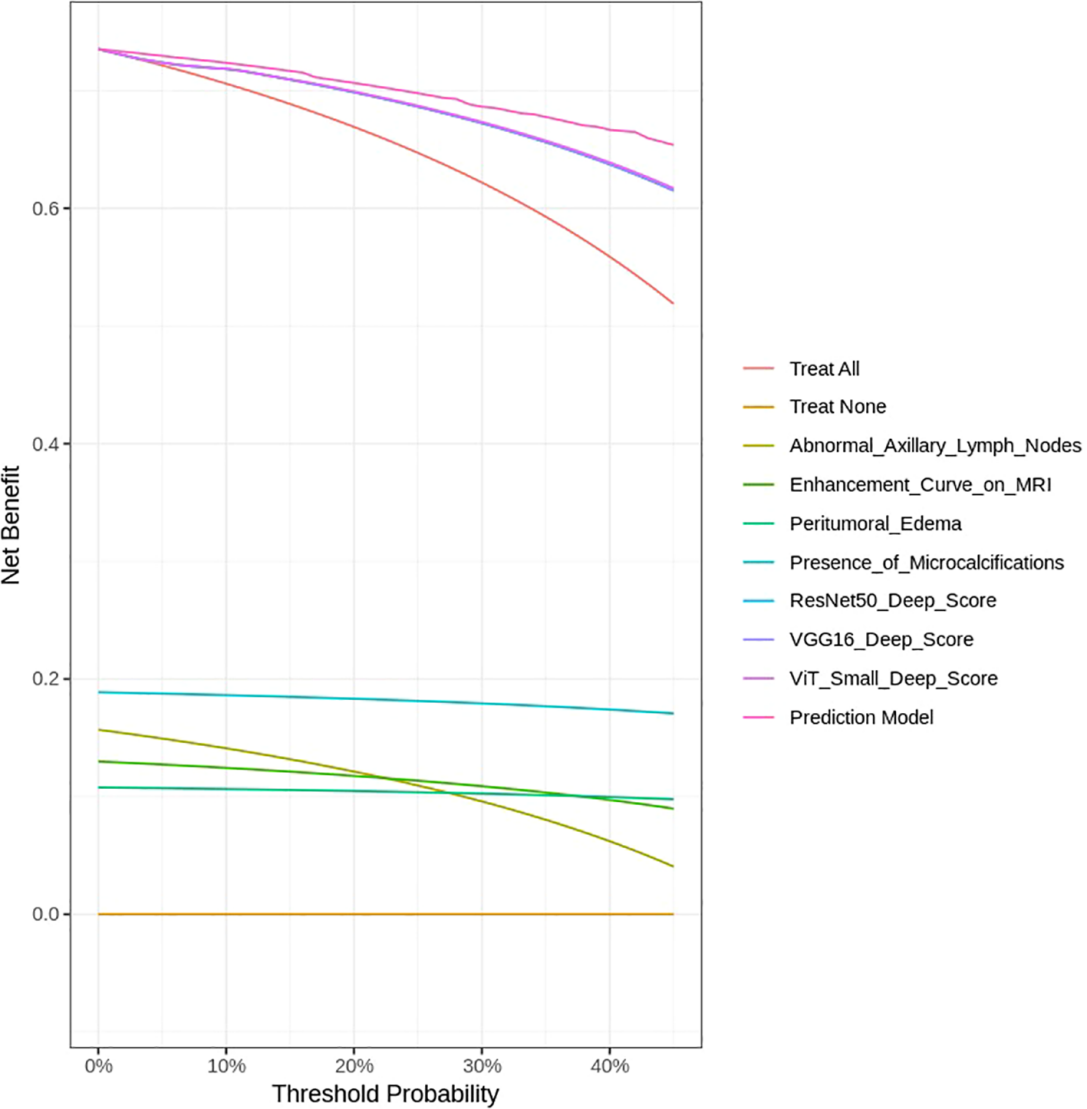
DCA for assessing the clinical utility of predictive models.

As shown in **Figure 14**, the Prediction Model that integrates deep learning features from ResNet50, VGG16, and ViT-Small, along with clinical variables such as Abnormal Axillary Lymph Nodes, Enhancement Curve on MRI, Peritumoral Edema, and Presence of Microcalcifications, consistently provides the highest net benefit across a range of threshold probabilities. This indicates that the integrated model outperforms individual features in guiding clinical decisions regarding HER2 classification in breast cancer.

Notably, the Prediction Model shows a clear advantage over treating all patients (labeled as Treat All) and treating none (Treat None) across the threshold range, highlighting its potential for personalized treatment planning. The deep learning models, particularly VGG16 and ViT-Small, also show strong net benefits, though they do not surpass the Prediction Model. In contrast, clinical features such as Abnormal Axillary Lymph Nodes and Enhancement Curve on MRI provide a moderate net benefit, while Peritumoral Edema and Presence of Microcalcifications offer limited utility when considered in isolation.

These findings underscore the importance of combining advanced imaging data with clinical features to develop more robust and clinically applicable models. The DCA curve demonstrates that the Prediction Model, which integrates these multiple sources of information, provides the best clinical utility for HER2 classification, supporting its potential for improving decision-making in breast cancer management. Although the integrated model achieved an AUC of 0.94 with strong calibration and DCA performance, these results are based on internal validation. External validation in independent cohorts is required to fully confirm the generalizability of the framework.

## 4 Discussion

This study developed a comprehensive approach to predicting HER2 expression in breast cancer by integrating deep learning-based feature extraction from multi-sequence breast MRI scans with clinical data through a nomogram-based predictive model. Our methodology is notable for its incorporation of four distinct deep learning architectures—ResNet50, VGG16, EfficientNet-B0, and Vision Transformer (ViT-Small)—which were leveraged to extract high-dimensional features from breast MRI images. These deep learning models, in combination with carefully selected clinical variables, provide a powerful framework for HER2 classification, achieving a notable AUC of 0.94 in predicting HER2 positivity. The robustness of our model is supported by validation in a multicenter dataset of 6,438 patients, which adds external validity to the results. This work represents a significant step forward in automating and improving the accuracy of HER2 classification, which is critical for guiding personalized treatment strategies in breast cancer.

An important strength of this study is that the reduced feature set retained by ICC and LASSO included descriptors with established biological and clinical relevance. Features such as washout enhancement, peritumoral edema, irregular margins, and nodal involvement have all been linked to aggressive HER2-positive disease in prior imaging and radiogenomic studies. The alignment of our model-selected features with these known biomarkers enhances interpretability, suggesting that the framework captures meaningful mechanistic correlates of HER2 amplification rather than relying solely on abstract computational representations.

When compared to other studies in the field, several similarities and differences emerge in terms of methodology, machine learning models used, and predictive performance. For instance, Qin et al. (17) similarly aimed to predict HER2 positivity using a machine learning model based on both imaging and clinical features. However, unlike our study, which employed deep learning models for feature extraction, Qin utilized Extreme Gradient Boosting (XGBoost) combined with an Artificial Neural Network (ANN) for feature selection and prediction. The ANN model in study of Qin et al. demonstrated an AUC of 0.853, which is comparable to the performance of our deep learning-based models on similar tasks. However, our approach, using deep learning models for direct feature extraction, offers a more automated and potentially more accurate alternative, particularly when dealing with high-dimensional MRI data (17).

In the study by Miglietta et al. (18), machine learning was employed to predict the conversion of HER2–0 breast cancer to HER2-low metastases using XGBoost and a support vector machine ensemble. While their focus was on metastatic conversions, the results underscore the utility of machine learning in HER2-related classification tasks. They achieved a balanced accuracy of 64%, a sensitivity of 75%, and a specificity of 53%, which, while promising, lags behind the performance of our integrated model, which demonstrated an AUC of 0.94. The comparative strength of our model lies in the inclusion of a variety of deep learning models and a large, diverse dataset, offering improved generalizability and higher discriminatory power (18).

Bitencourt et al. (1) also employed machine learning in conjunction with radiomics to predict HER2 status in HER2-overexpressing breast cancer patients receiving neoadjuvant chemotherapy (NAC). This study highlighted the importance of combining clinical and radiomic features from MRI scans, achieving a high AUC of 0.97 in predicting HER2 heterogeneity and a diagnostic accuracy of 83.9% for predicting pathologic complete response (pCR). Our study builds on this by incorporating multi-sequence MRI data and applying deep learning to automatically extract hierarchical features from the images, while their approach manually selects radiomic features based on correlation analysis. Our results, particularly the AUC of 0.94, are in line with their findings but are made more efficient and less prone to human bias due to the use of deep learning for feature extraction (1).

The work by Wu et al. (19) focused on deep learning for predicting HER2 status and treatment efficacy in gastric adenocarcinoma (GAC). Their study used convolutional neural networks (CNN) to predict HER2 amplification with an AUC of 0.847 and a higher AUC of 0.903 for predicting HER2 2+ status. This approach demonstrates the broader applicability of deep learning in predicting HER2 status across cancer types, providing a useful comparison to our breast cancer-focused study. While their CNN model had strong performance, it did not incorporate multi-sequence imaging or clinical variables as comprehensively as our study, limiting its interpretability and external applicability (19). In another study by Luo et al. (4), radiomics features extracted from multi-sequence breast MRI were utilized to predict HER2 expression in invasive ductal carcinoma (IDC), achieving AUC values of 0.777 for classifying HER2-positive from HER2-negative cases. While their study employed traditional radiomics techniques, our study benefited from a deeper feature extraction approach using deep learning, which automatically captures complex image patterns without relying on manual feature selection. As a result, our model outperformed traditional radiomics approaches, highlighting the advantage of using deep learning over handcrafted features in complex imaging datasets.

Moreover, the study by Yan et al. (3) used ultrasound radiomics and clinical features to predict HER2 status in breast cancer patients with indeterminate HER2-2+ immunohistochemical results, achieving an AUC of 0.860 with logistic regression. This study shares similarities with ours in utilizing non-invasive imaging modalities (ultrasound vs. MRI). However, our study outperforms theirs by leveraging more advanced deep learning models and integrating multi-sequence MRI data, providing a richer, more detailed set of features for accurate HER2 classification.

In comparison, our study’s use of deep learning for feature extraction stands out for its automated, high-dimensional approach, which reduces the potential for human bias and error compared to the more manual radiomics methods used in other studies. Furthermore, while other studies, such as Boulmaiz et al. (20), employed machine learning techniques (including random forest and LightGBM) for predicting HER2 status in breast cancer, our model achieved superior performance by combining multi-sequence imaging and clinical data with deep learning, resulting in a higher AUC and more reliable predictions.

Lastly, Mastrantoni et al. (21) and Xu et al. (22) both used machine learning models to predict HER2 status in different cancer types (early breast cancer and gastric adenocarcinoma, respectively). Their use of different types of imaging (CT and ultrasound) and various machine learning models provides insights into the flexibility of AI tools across different cancers. However, their models did not demonstrate the same level of performance as our deep learning-based approach using MRI, where higher-dimensional data provided richer feature sets for prediction.

A key limitation of this study is the absence of external validation. While internal validation with 1,000 bootstrap resamples demonstrated robust performance, the lack of testing on independent cohorts limits the immediate generalizability of the results. Future work will focus on validating the model in external multicenter datasets and prospective studies to confirm its clinical applicability. It should be noted that the prevalence of HER2-positive cases in our final cohort (53.6%) was higher than the 15–20% typically observed in the general breast cancer population. This enrichment reflects the strict inclusion criteria requiring complete multi-sequence MRI and confirmed HER2 status, which are more frequently fulfilled in HER2-positive patients who undergo comprehensive imaging and biomarker testing in clinical practice. Although measures such as multicenter recruitment, reproducibility filtering (ICC ≥ 0.9), LASSO regression, and bootstrap validation were implemented to mitigate bias, future external validation in population-based and prospective cohorts will be essential to further confirm generalizability.

Although a substantial number of patients were excluded due to missing or poor-quality data, comparison with the included cohort showed no systematic demographic or clinical differences (**Supplementary Table S3**), reducing concern for major selection bias.

## 5 Conclusion

This study’s results support the growing body of evidence that deep learning-based models, when combined with multi-sequence imaging and clinical data, offer superior performance for HER2 classification in breast cancer. In comparison with other studies, our approach achieved higher AUCs and demonstrated more robust performance across diverse datasets, suggesting that deep learning holds significant promise for improving the accuracy and efficiency of HER2 expression status prediction, with potential clinical applications in personalized oncology. Further studies and validation across different clinical settings and imaging protocols will be crucial for translating these findings into routine clinical practice.

## Data Availability

The data analyzed in this study is subject to the following licenses/restrictions: The datasets generated and analyzed during the current study are available from the corresponding author upon reasonable request. Requests to access these datasets should be directed to Yun Zhao, 11618176@zju.edu.cn.
