# Supplementary material for: Novel deep learning-based prediction of HER2 expression in breast cancer using multimodal MRI, nomogram, and decision curve analysis
Source: Front Oncol. 2025 Oct 29;15:1593033. doi: 10.3389/fonc.2025.1593033 (PMC12605381; doi:10.3389/fonc.2025.1593033)
Supplement: Supplementary file 1 [file DataSheet1.docx]

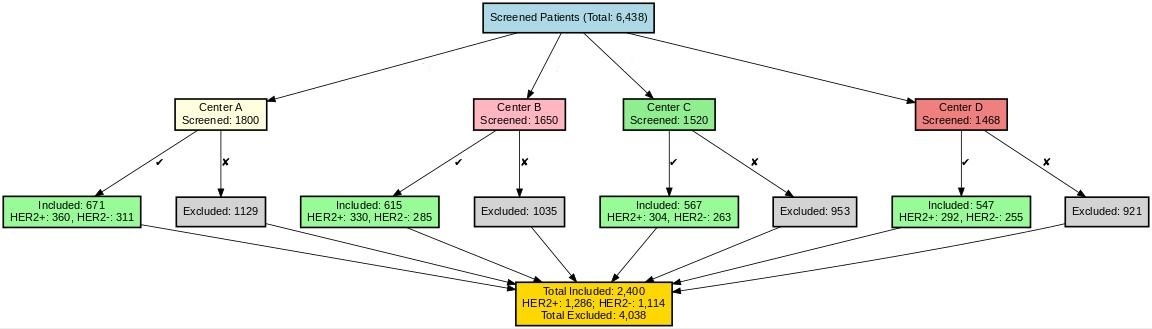


**Supplementary Figure S1.** Patient enrollment and eligibility flow diagram. Among 6,438 patients screened across four tertiary centers, 4,038 were excluded due to incomplete MRI sequences, missing or indeterminate HER2 status, low-quality imaging artifacts, or prior neoadjuvant therapy. The final analyzed cohort comprised 2,400 patients (HER2-positive: 1,286; HER2-negative: 1,114).
