# Supplementary material for: Novel deep learning-based prediction of HER2 expression in breast cancer using multimodal MRI, nomogram, and decision curve analysis
Source: Front Oncol. 2025 Oct 29;15:1593033. doi: 10.3389/fonc.2025.1593033 (PMC12605381; doi:10.3389/fonc.2025.1593033)
Supplement: Supplementary file 2 [file Table1.docx]

**Supplementary Results: Fine-Tuned vs. Non-Fine-Tuned Models**

Pilot experiments were conducted to compare fine-tuned versus non-fine-tuned (frozen-weight) strategies across the four pretrained models (ResNet50, VGG16, EfficientNet-B0, ViT-Small). Fine-tuned models achieved very high training accuracy but consistently overfit, with reduced external validation AUCs and poorer calibration. Frozen-weight models, while showing slightly lower training accuracy, provided superior generalization across centers.

**Supplementary Table S1. Performance comparison of fine-tuned vs. non-fine-tuned (feature extraction only) models on HER2 classification.**

| Model | Strategy | AUC (95% CI) | Precision | Recall | F1-score |
| --- | --- | --- | --- | --- | --- |
| ResNet50 | Fine-tuned | 0.85 (0.82–0.87) | 0.78 | 0.74 | 0.76 |
|  | Frozen (no tuning) | 0.87 (0.84–0.89) | 0.82 | 0.77 | 0.79 |
| VGG16 | Fine-tuned | 0.86 (0.83–0.88) | 0.80 | 0.76 | 0.78 |
|  | Frozen (no tuning) | 0.89 (0.86–0.91) | 0.83 | 0.80 | 0.81 |
| EfficientNet-B0 | Fine-tuned | 0.82 (0.79–0.85) | 0.75 | 0.71 | 0.73 |
|  | Frozen (no tuning) | 0.85 (0.82–0.87) | 0.79 | 0.75 | 0.77 |
| ViT-Small | Fine-tuned | 0.88 (0.85–0.90) | 0.83 | 0.78 | 0.80 |
|  | Frozen (no tuning) | 0.91 (0.89–0.93) | 0.86 | 0.82 | 0.84 |
