# Supplementary material for: Novel deep learning-based prediction of HER2 expression in breast cancer using multimodal MRI, nomogram, and decision curve analysis
Source: Front Oncol. 2025 Oct 29;15:1593033. doi: 10.3389/fonc.2025.1593033 (PMC12605381; doi:10.3389/fonc.2025.1593033)
Supplement: Supplementary file 3 [file Table2.docx]

**Supplementary Table S2.** Baseline characteristics across four centers (A–D).

| Variable | Center A | Center B | Center C | Center D | p-value |
| --- | --- | --- | --- | --- | --- |
| Age (mean ± SD) | 55.1 ± 10.8 | 54.9 ± 11.0 | 55.3 ± 11.1 | 54.6 ± 10.7 | 0.72 |
| Menopausal status (%) | 64.0 / 36.0 | 63.5 / 36.5 | 65.2 / 34.8 | 64.1 / 35.9 | 0.81 |
| Tumor size (cm) | 2.6 ± 1.0 | 2.5 ± 1.1 | 2.7 ± 1.0 | 2.6 ± 1.1 | 0.65 |
| Histological type (%) | 91.5 / 8.5 | 92.0 / 8.0 | 91.0 / 9.0 | 90.8 / 9.2 | 0.88 |
| Molecular subtype (%) | 58.0 / 30.5 / 11.5 | 57.2 / 31.0 / 11.8 | 57.8 / 30.1 / 12.1 | 56.9 / 31.3 / 11.8 | 0.93 |
