# Supplementary material for: Novel deep learning-based prediction of HER2 expression in breast cancer using multimodal MRI, nomogram, and decision curve analysis
Source: Front Oncol. 2025 Oct 29;15:1593033. doi: 10.3389/fonc.2025.1593033 (PMC12605381; doi:10.3389/fonc.2025.1593033)
Supplement: Supplementary file 4 [file Table3.docx]

### **Supplementary Table S3.** Comparison of baseline characteristics between included (n = 2,400) and excluded (n = 4,038) patients.

| Variable | Included (n=2400) | Excluded (n=4038) | *p*-value |
| --- | --- | --- | --- |
| Age (years, mean Â± SD) | 55.0 ± 11.0 | 54.7 ± 11.2 | 0.41 |
| Menopausal status (Postmenopausal %) | 64.00% | 62.90% | 0.32 |
| Tumor size (cm, mean Â± SD) | 2.6 ± 1.0 | 2.4 ± 1.1 | 0.08 |
| Histological subtype (IDC %) | 91.50% | 90.80% | 0.27 |
| HER2 positive (%) | 53.60% | 52.10% | 0.35 |
